# Bibliographical

**Published:** 1892-02

**Authors:** 


### Bibliographical.

Dental Medicine.—A Manual of Dental Materia Medica and Therapeutics, by Ferdinand J. S. Gorgas, A.M., M.D., D.D.S., editor of "Harris' Principles and Practice of Dentistry" and "Harris' Dictionary of Medical Terminology and Dental Surgery," Professor of the Principles of Dental Science, Dental Surgery, etc., in the University of Maryland, Baltimore. Fourth edition, revised and enlarged. Published by P. Blackiston, Son & Co., No. 1012 Walnut st., Philadelphia, Pa.

"In presenting a fourth edition of the 'Dental Medicine' to the dental profession the author desires to express his grateful appreciation for the favors with which each of the preceding editions has been received, and the kind notices they have elicited."

The enlargement of this fourth edition has been necessary in order to bring the work up to the present status of dental materia medica and therapeutics. Considerable matter has been added to diagnosis of the mouth, the different remedial agents, the various substances classed as dental materia medica with their medicinal properties, action, dental use and mode of application, among the number arsenious acid, carbolic acid, aromatic sulphuric acid, tannic acid, chloroform, nitrous oxide, chloral, antipyrene, antifebrin, bichloride of mercury, peroxide of hydrogen, creolin, chloride of methyl, sulphonal, etc., etc.

A new chapter has been added on the use of antiseptics in dental practice which includes the sterilization of dental and surgical instruments, also a list of new antiseptics, disinfectants, germicides, and hypnotics, etc., together with a number of new and valuable formulæ.

Lengthy notice has already been made of a former edition of this book, so that little remains to be said except that this present editon is a revision and enlargement of the third. The name of the author of this work, so well known to every dental student throughout the country, and the necessity for the issue of a fourth edition, combined with a knowledge that every copy of each of the former editions had been disposed of some months before the subsequent edition was ready for publication, are sufficient guarantee of the value of this book to all interested in the practice and study of dentistry. So much has been added to this fourth edition that a glance at the table of contents and the general index will at once convince them of the value of this work as a text-book and book of reference. It is neatly bound in cloth and the tpye is remarkably clear. Every dental student will find it to his interest to obtain a copy.

